# Frequency dependence of coordinated pupil and eye movements for binocular disparity tracking

**DOI:** 10.3389/fneur.2023.1081084

**Published:** 2023-06-15

**Authors:** Carey D. Balaban, Neil S. Nayak, Erin C. Williams, Alexander Kiderman, Michael E. Hoffer

**Affiliations:** ^1^Departments of Otolaryngology, Neurobiology, Communication Sciences & Disorders, Bioengineering, and Mechanical Engineering & Materials Science, University of Pittsburgh, Pittsburgh, PA, United States; ^2^Silverstein Institute, Sarasota, FL, United States; ^3^Department of Otolaryngology, University of Miami, Miami, FL, United States; ^4^Neurolign USA, Inc., Pittsburgh, PA, United States; ^5^Neurological Surgery and Sports Performance and Wellness Institute, University of Miami, Miami, FL, United States

**Keywords:** vergence, near response, binocular disparity stimuli, smooth pursuit, coordination, synkinesis

## Abstract

**Introduction:**

Coordinated alignment of the eyes during gaze fixation and eye movements are an important component of normal visual function. We have previously described the coordinated behavior of convergence eye movements and pupillary responses using a 0.1  Hz binocular disparity-driven sine profile and a step profile. The goal of this publication is to further characterize ocular vergence-pupil size coordination over a wider range of frequencies of ocular disparity stimulation in normal subjects.

**Methods:**

Binocular disparity stimulation is generated by presentation of independent targets to each eye on a virtual reality display, while eye movements and pupil size are measured by an embedded video-oculography system. This design allows us to study two complimentary analyses of this motion relationship. First, a macroscale analysis describes the vergence angle of the eyes in response to binocular disparity target movement and pupil area as a function of the observed vergence response. Second, a microscale analysis performs a piecewise linear decomposition of the vergence angle and pupil relationship to permit more nuanced findings.

**Results:**

These analyses identified three main features of controlled coupling of pupil and convergence eye movements. First, a near response relationship operates with increasing prevalence during convergence (relative to the “baseline” angle); the coupling is higher with increased convergence in this range. Second, the prevalence of “near response”-type coupling decreases monotonically in the diverging direction; the decrease persists after the targets move (converge back) from maximum divergence toward the baseline positions, with a minimum prevalence of near response segments near the baseline target position. Third, an opposite polarity pupil response is infrequent, but tends to be more prevalent when the vergence angles are at maximum convergence or divergence for a sinusoidal binocular disparity task.

**Discussion:**

We suggest that the latter response is an exploratory “range-validation” when binocular disparity is relatively constant. In a broader sense, these findings describe operating characteristics of the near response in normal subjects and form a basis for quantitative assessments of function in conditions such as convergence insufficiency and mild traumatic brain injury.

## Introduction

**Figure 1:**
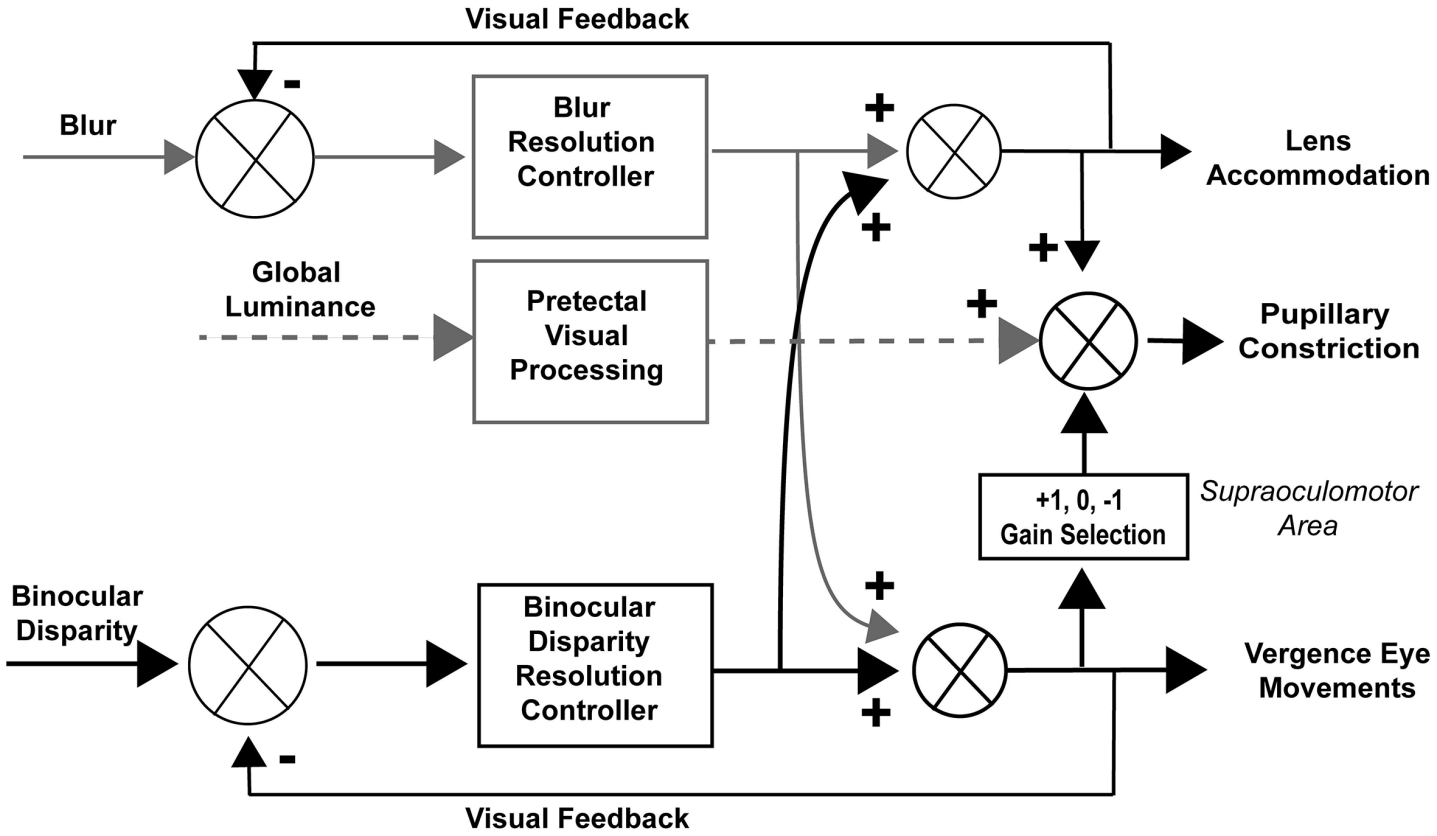
Reproduced with permission from Balaban et al. (2).

Coordinated regulation of ocular convergence, lens accommodation and pupil size is a hallmark of the well-known near response (1). Figure 1 shows a schematic diagram of mechanisms for coordination of pupillary and convergence eye movements from our previous publication (2). Three stimulus-related input signals for binocular control are shown: blur, global illumination, and binocular disparity. The near response is described classically as a coordination of convergence, pupillary constriction and lens accommodation, with pupillary constriction accompanying ocular convergence and dilation accompanying ocular divergence. However, microscale analysis revealed that the relationship is sometimes reversed transiently, with pupillary dilation accompanying ocular convergence or pupillary constriction accompanying ocular divergence. This communication examines the similarity of these responses across binocular disparity vergence stimulus frequencies. Binocular disparity is modified independently by moving monocular targets on a virtual reality display, while eye movements and pupil size are measured by an embedded video-oculography system.

Our earlier publication (2) analyzed patterns of pupillary activity during binocular disparity convergence in normal subjects with two binocular disparity stimulus profiles, a 0.1 Hz sine profile and a step profile. Two approaches were used to identify properties of eye-pupil coordination. A macroscale analysis estimated parameters of transfer functions that describe (1) the vergence angle of the eyes in response to binocular disparity target movement and (2) pupil area as a function of that observed ocular vergence angle. To account for asymmetries, the analysis calculated separate parameters in the converging versus diverging directions for eye movements and for accompanying pupil responses. A second, microscale analysis conducted a piecewise linear decomposition of the relationship between pupil area and the vergence angle, in order to identify coordination on a more granular time scale. The goal of this work is to extend these analyses of ocular vergence-pupil size coordination over a wider range of frequencies of ocular disparity stimulation to test if there are frequency-related differences in normal subjects and establish baselines for test parameters in clinical settings for quantitative assessment of convergence insufficiencies.

## Materials and methods

Subjects were recruited at University of Miami (23 subjects: 7 males, 16 females). Subjects ranged in age from 19 to 52  years [31.1 ± 7.7 years]. Informed consent was obtained. Potential subjects were excluded if they had history of a central processing disorder, impaired vision without corrective lenses (maximum of 20/60 uncorrected), moderate to severe hearing loss (>55 dB PTA, <50% word identification), vestibular disorders, history of ear surgery (other than myringotomy with or without tube placement). Pregnant women, prisoners, and individuals unable to consent were also excluded. The project was approved by the IRB at the University of Miami and analysis of de-identified data was approved by the IRB at the University of Pittsburgh.

Binocular disparity vergence eye movement performance was tested quantitatively with clinical eye tracking system within a portable 3D head mounted display system (Neurolign Dx100, formerly marketed as Neuro Kinetics I-PAS^™^; I-Portal^®^ Portable Assessment System, Neurolign United States, Pittsburgh, PA, United States). Each eye viewed an independent circular portion of a 1,920 × 1,080 pixel stimulus display that subtended a 60 degree diagonal field of view. Subjects adjusted the focus of the video image for each eye across an available ±4 diopter range. The effective viewing distance is e between 1 and 1.5 meters. Video-based eye tracking was performed under continuous 940 nm infrared illumination at a sampling rate of 100 Hz. Pupils were identified by characteristic luminance boundaries. The pupil area was measured from each image at a resolution of 139 pixels/mm^2^. The instantaneous eye position was calculated from the centroid of the identified pupil area. Horizontal (±30 degree range) and vertical (±20 degree range) eye tracking spatial resolution was on the order of 0.02°, while spatial resolution for torsional eye movement (±10 degree range) was <0.1°.

Neurolign VEST^™^ software was used to control testing and data collection. All stimuli were rendered in the virtual environment that was created by the enclosed video display, with synchronization of the stimulus refresh rates and the eye tracking sampling rate. Eye movement recordings were calibrated for a series of conjugate horizontal and vertical gaze shifts, using spot targets subtending approximately 0.1 degree of visual angle. Vergence angle is represented in degrees relative to zero at initial fixation. Normal consensual pupil responses were confirmed during the neurological examination and tested quantitatively as described in previous publications (2).

Targets for the disparity vergence tasks were a white square with red center that covered approximately 0.1 degrees visual angle of each eye (field luminance: 0.05–0.06 cd/m^2^), presented to each eye on the headset. For disparity step task, targets moved at 4 s intervals between a disparity requiring a 1.5° convergence and a disparity requiring a 1.5° divergence for 5 cycles. For disparity pursuit, the disparity moved sinusoidally (divergence first) in the following order, with a pause between each trial: (1) 0.1 Hz sine (3 cycles), (2) 0.1 Hz Step (4 cycles), (3) 0.2 Hz sine (2 cycles), (3) 0.07 Hz sine (2 cycles), (4) 0.13 Hz sine (2 cycles), (5) 0.17 Hz sine (2 cycles), (6) 0.07 Hz sine (2 cycles), (7) 0.13 Hz sine (2 cycles), (8) 0.1 Hz sine (2 cycles), (9) 0.17 Hz sine (2 cycles), and (10) 0.2 Hz sine (2 cycles), also a ± 1.5 deg. binocular disparity magnitude.

Quantitative analysis of coordinated eye movements and pupil size was based upon published models and algorithms (2). Calibrated data were exported and analyzed with MATLAB (MathWorks, Natick, MA) and SPSS Statistics 24 (IBM). The data are referenced to zero vergence and zero pupil baseline by subtracting mean pupil area as the initial step of estimating dynamic properties. By convention, positive vergence angle is defined as converged relative to baseline and negative angle is defined as diverged.

Cross spectral analyses were used to assess coherence of the consensual pupil size fluctuations. They were conducted using the standard signal toolbox functions “mscohere.m,” “cpsd.m,” and “pwelch.m” in MATLAB. Two analytic methods were implemented in MATLAB: (1) macroanalysis by estimating parameters of a linear global response model and (2) microanalysis by piecewise linear segmentation of the pupil size-vergence angle relationship. The convergence angle of eye movements in the disparity step task were modeled as the weighted sum of first order high and low pass representations of the vergence target position with a processing delay. Nonlinear least squares regression (“lsqnonlin.m” function in MATLAB) was used to estimate parameters for the vergence disparity response as a weighted sum of high pass ( $\frac{K_{vh}se^{-t_{v}s}}{s+1}\;)\;$ and low pass ( $\frac{K_{vl}e^{-t_{v}s}}{0.25s+1}$ ) processes, with delay t_v_ and gains K_vh_ (high pass process) and K_vl_, (low pass process), respectively. The delay parameter accounts for the reaction time to the binocular disparity step stimulus; it was set at zero for the binocular disparity pursuit task. The fixed time constants of 0.25 s for the low pass vergence eye movement process and 1 s for the high pass response provided a robust fit for both the disparity step responses and sinusoidal disparity stimuli in our earlier study of normal subjects (2). Initial gain parameters for optimization were set at unity. Pupil dynamics were fitted from the vergence data by a transfer function for pupil motion, $\frac{K_{p}e^{-t_{p}s}}{0.28s+1}$ , with delay t_p_ and gain K_p_, which estimates the near response sensitivity directly. The time constant (0.28 s) for pupil dynamics was adopted from the internal control model in Sun et al. (3). The initial condition for the pupil delay parameter was set at 0.2 s, consistent with both Sun et al. (3) and experimental confirmation in normal subjects (2) and initial gain parameters for optimization were set at unity. Symmetry was tested by fitting separate gains for half-cycles of convergence versus divergence and for half-cycles of pupil constriction versus dilatation.

For the microscale analysis, a modified Gath-Geva clustering algorithm (4) performed an objective fuzzy segmentation of the time series into 15 segments with homogeneous properties. The first step of this algorithm is a principal component decomposition to identify a component that represents the instantaneous pupillary area relative to instantaneous vergence angle. A subsequent clustering algorithm decomposes the data into linear segments, based upon both metrics of the homogeneity of those segments and the fuzzy sets that are used to represent the segments in time.

## Results

**Figure 2:**
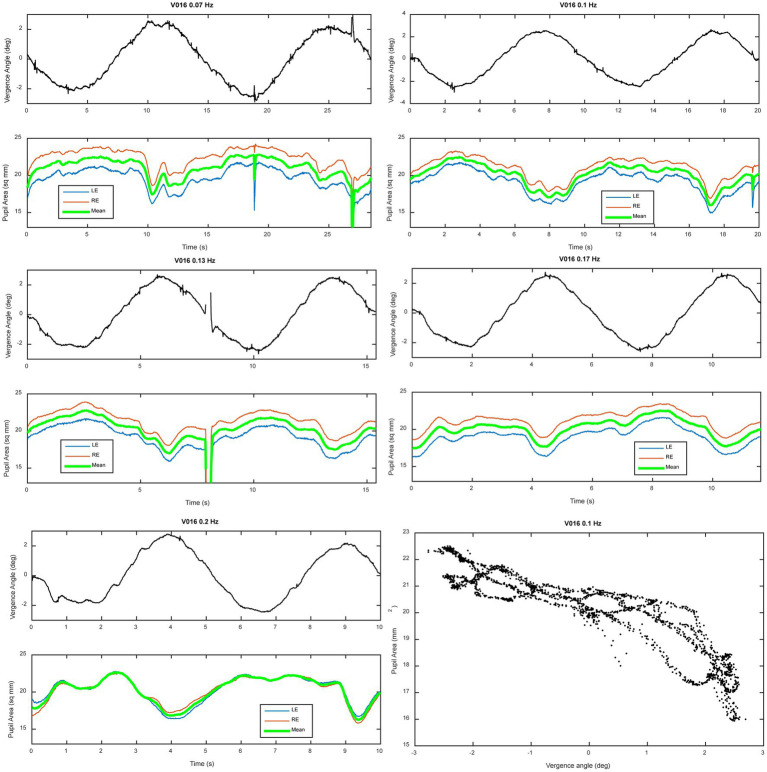
Vergence eye movement and average pupil size adjustments during binocular disparity pursuit testing. A representative example of a series of tests at different frequencies is shown for a representative subject. Two panels are shown for each frequency; the upper panel shows vergence angle (convention: convergence from initial angle is positive), while the lower panel shows the simultaneous recordings mean (green), left (blue) and right (right) pupil sizes. The time bases of the graphs are set to show two cycles of stimulation at each frequency. The lower right panel shows a plot of the instantaneous pupil size as a function of instantaneous vergence angle; the prominent quasi-linear relationship shows the strong co-regulation of pupil size and vergence angle in normal subjects (the “near response”). Notice the steeper slope of the relationship during convergence (vergence angle >0) relative to divergence (vergence angle <0).

Figure 2 shows data traces from a typical normal subject during sinusoidal binocular disparity tracking. The coordinated eye and pupil movements are accompanied by the perception of the target periodically receding, then approaching the bridge of the nose. The time scales on the graphs are adjusted to display two response cycles at each frequency, with the vergence trace (black) in the upper panel and concurrent pupil size responses in the lower panel. By convention, a positive convergence angle indicates convergence and a negative angle indicates divergence. Note that the vergence response is smooth, symmetric, and sinusoidal across the frequency range (0.07–0.2 Hz), with a slight initiation delay during the first quarter cycle of stimulation. The symmetry is confirmed by the equivalence of converging and diverging eye movement gains for both the low pass and high pass model components (Table 1, no significant differences by repeated measures ANOVA, Bonferroni adjusted criteria). These parameters accounted for 81.6–88.6% of the variance across the frequency range (Table 1, *R*^2^ column). Repeated measures ANOVA showed no significant differences across frequencies in the vergence eye movement model parameters (high pass gain diverged, high pass gain converged, low pass gain diverged, low pass gain converged). When age (range 19–52 years) was included as a covariate in repeated measure ANOVA, there were neither a significant age main effect nor significant age interaction effects for any metric of eye or pupil responses. Hence, the dynamic control of binocular disparity vergence eye movements was invariant for sinusoidal profiles between 0.07 and 0.20 Hz for stimuli requiring ±1.5° vergence tracking (convergence positive by convention).

**Table 1:** Vergence Eye Movement Fit and Symmetry re: Disparity Stimulus (disparity sine).

Frequency	R^2^ (mean ± SE)	Low pass		High pass	
		Converging	Diverging	Converging	Diverging
0.07 Hz	0.886 ± 0.020	1.632 ± 0.074°	1.658 ± 0.094°	0.528 ± 0.177°	0.384 ± 0.191°
0.10 Hz	0.849 ± 0.034	1.564 ± 0.094°	1.601 ± 0.088°	0.597 ± 0.189°°	0.306 ± 0.114°
0.13 Hz	0.878 ± 0.025	1.509 ± 0.076°	1.487 ± 0.087°	0.619 ± 0.121°	0. 565 ± 0.147°
0.17 Hz	0.852 ± 0.038	1.495 ± 0.160°	1.487 ± 0.159°	0.401 ± 0.201°	0.402 ± 0.112°
0.20 Hz	0.816 ± 0.041	1.436 ± 0.101°	1.413 ± 0.164°	0.676 ± 0.133°	0.391 ± 0.168°

The traces in Figure 2 also show two notable features of pupillary area regulation during the disparity-induced vergence eye movements. First, the left and right pupil responses are consensual at all frequencies; the left pupil (green trace) and right pupil (red trace) size changes are parallel at all frequencies. Second, the pupil size response is asymmetric when the eyes are converged relative to zero position versus diverged from that position. Specifically, the responses appear blunted (and partially rectified) in the diverging versus converging directions. This asymmetry can be seen clearly in the lower right panel of Figure 2 by the shallower slope of the instantaneous pupil size-vergence angle relationship when vergence angle is less than zero (eyes diverged relative to baseline angle for the test).

**Figure 3:**
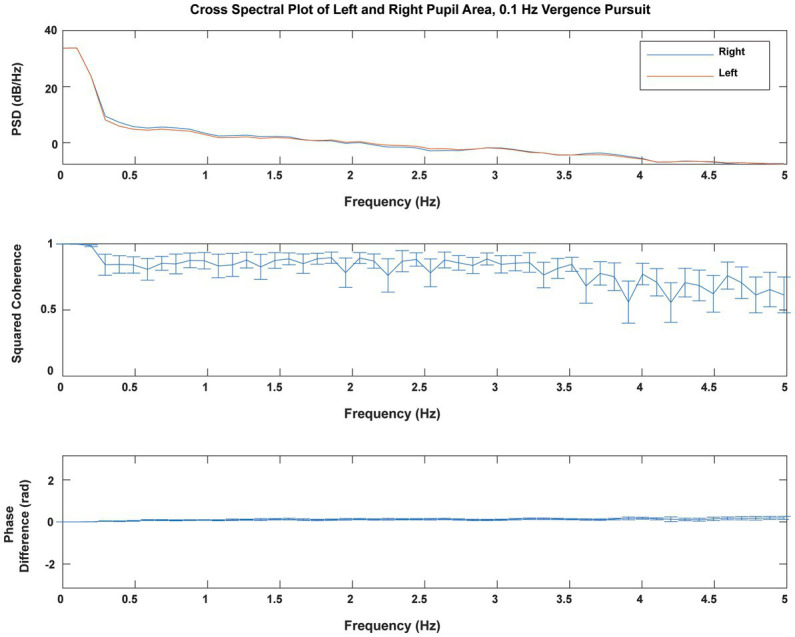
Cross spectral analysis of simultaneous right and left pupil areas during 0.1 Hz binocular disparity vergence pursuit. There was little power above 5 Hz (one-twentieth of the sampling rate). The average power spectra (upper panel) for the right pupil (blue) and left pupil (red) areas were virtually identical. The squared coherence (shown ±2 standard errors) remained above 0.8 for frequencies below 3 Hz and remained above 0.6 at 5 Hz. The cross-spectral phase difference was negligible. These analyses illustrate the profound consensual nature of the neural control of the pupils.

The consensual fidelity of the left and right pupil responses was tested explicitly by cross spectral analysis of the responses of both eyes. Figure 3 shows the average coherence of pupil areas from both eyes of 23 subjects during sinusoidal binocular disparity vergence at 0.1 Hz. The power spectra of the right and left pupil responses were very similar (upper panel). The responses showed high squared coherence (>0.8) to greater than 3 Hz (middle panel), and it remained above 0.6 at 5 Hz. The cross-spectral phase difference was negligible across the 5 Hz range. These results did not vary across the different stimulus frequency conditions for binocular disparity vergence stimulation. Hence, the average pupil area was used for analyses of co-regulation of pupil size with vergence eye movements.

Despite the same eye movement behavior across frequencies, analyses based upon the model of pupil metrics revealed significant differences in pupil size regulation metrics as a function of frequency. The analyses came from two perspectives. A macroscale approach (2) was used to estimate delay, constricting sensitivity (gain), dilating sensitivity (gain), and linear drift parameters for a first order model that simulates pupil size from each vergence angle data trace. It provides a description of the steady state pupil response during periodic convergence. A microscale analysis approach is based on a piecewise linear decomposition of the relationship between instantaneous vergence angle and pupil size (relationship example in lower right panel of Figure 2).

**Figure 4:**
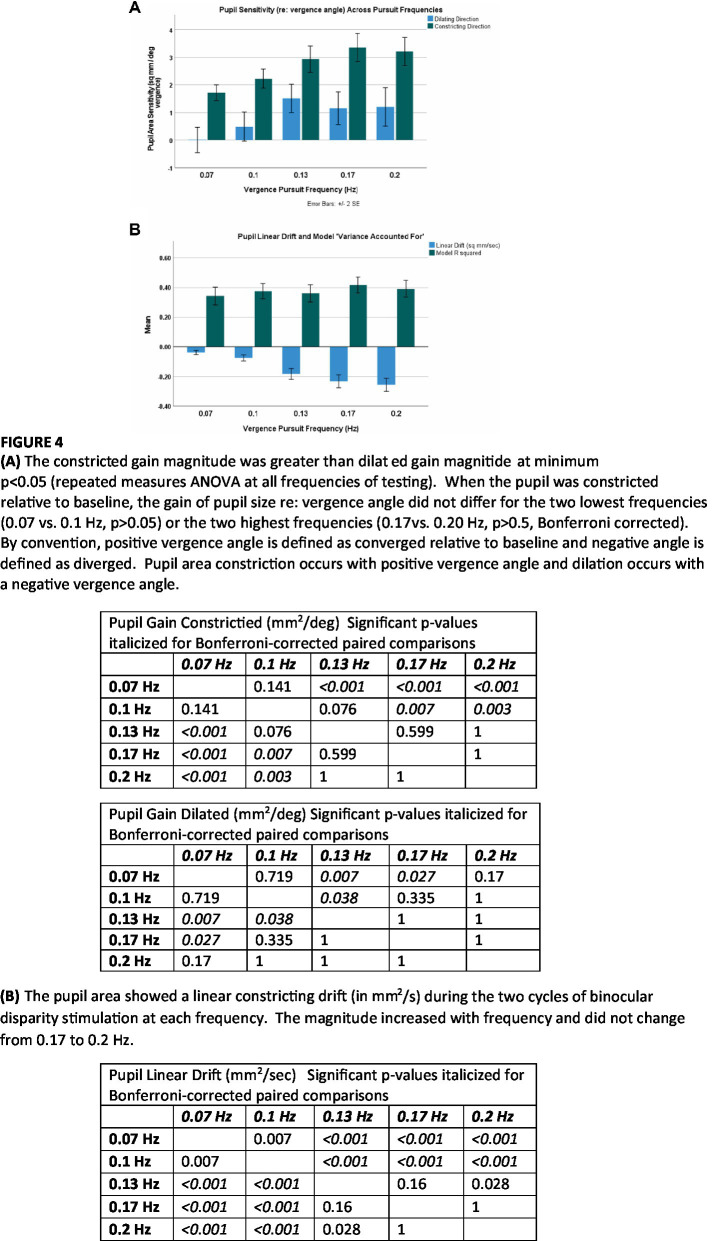


The results from the macroscale analysis are summarized in Figures 4A,B. The *R*^2^ values did not vary significantly across frequencies (Figure 4B). Properties of steady-state pupil size regulation varied significantly with the stimulus frequency (and peak velocity of the binocular disparity). The magnitudes of the pupil responses, expressed in mm^2^/deg. vergence, were greater at higher frequencies (repeated measures ANOVA and paired comparisons). The contricting gain (in mm^2^/deg. vergence) was significantly greater than zero at all frequencies, indicating significant constrictor component of the ‘near response’ for convergent eye positions during binocular disparity tracking. Further, the constricting gain was significantly greater than dilating gain (*p* < 0.002, repeated measures ANOVA, Bonferroni-corrected comparisons) at each frequency of testing. The dilating gain did not differ from zero at 0.07 or 0.10 Hz (two sided *t*-test, *p* > 0.05), but differed significantly from zero gain at 0.13, 0.17, and 0.20 Hz. There were no significant differences in the pupil delay parameter across frequencies (not shown, overall mean: 0.10 ± 0.05 s).

The linear drift component in pupil area (mm^2^/s) differed significantly from zero (*p* < 0.001) at all frequencies. The slope of a linear drift in perstimulus pupil area (in mm^2^/s) also increased significantly in magnitude with stimulus frequency (Figure 4B). The mean drift was also related linearly to the peak vergence velocity (peakVergVel) at each stimulus frequency; the linear relationship was −0.2772*peakVergVel +0.0784 with *r*^2^ = 0.94. The cumulative drift during the two cycles of stimulation was −1.1 mm^2^ at 0.07 Hz, −1.5 mm^2^ at 0.1 Hz, −2.8 mm^2^ at 0.13 Hz, −2.7 mm^2^ at 0.17 Hz and −2.5 mm^2^ at 0.2 Hz. Hence, responses to higher frequency (higher peak velocity) binocular disparity responses show two adjustments with changes in the frequency (or peak velocity) of binocular disparity stimulation: (1) increased dynamic pupillary responsiveness (in mm^2^/deg. vergence) and (2) a linear reduction in “baseline set point” for pupil area (expressed as a higher magnitude of constricting drift in mm^2^/second that scaled with peak vergence velocity).

**Figure 5:**
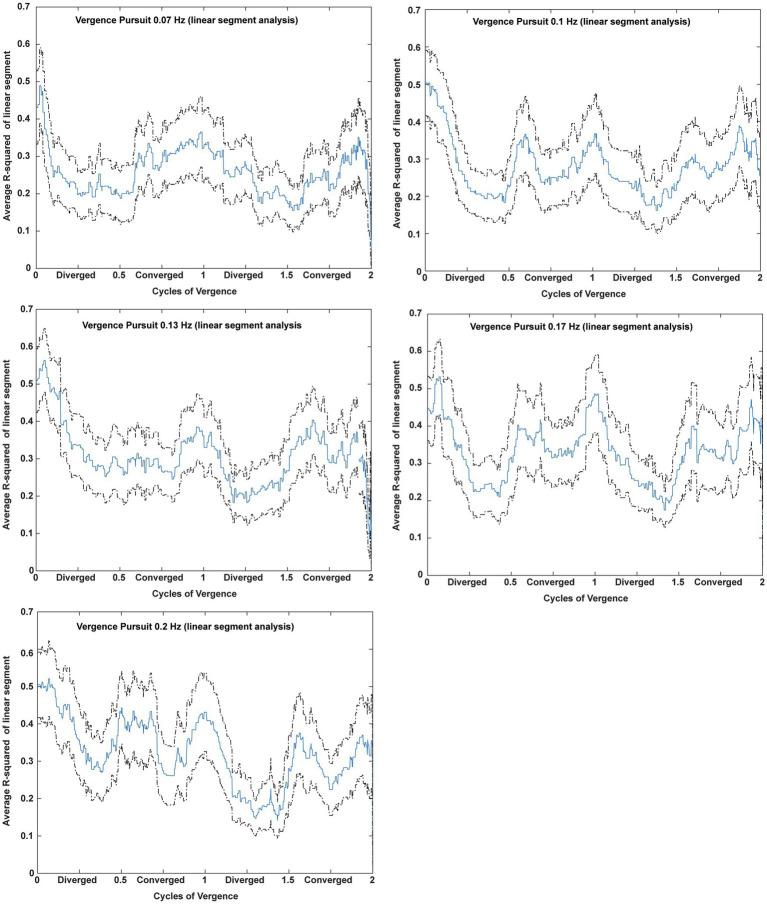
Average *R*^2^ value across subjects during two cycles of disparity vergence tracking. For each frequency of disparity vergence pursuit, the *R*^2^ estimate from the piecewise linear decompositions of the data were entered for each sample time from each subject. The data are displayed as a function of the cycle of convergence; cycle durations were different for each frequency. The mean (blue) and 2 standard error bands (black) show that the linear relationship is strongest during response initiation and the fidelity of the linear fits fluctuates with the location of the vergence target during the remainder of each session.

**Figure 6:**
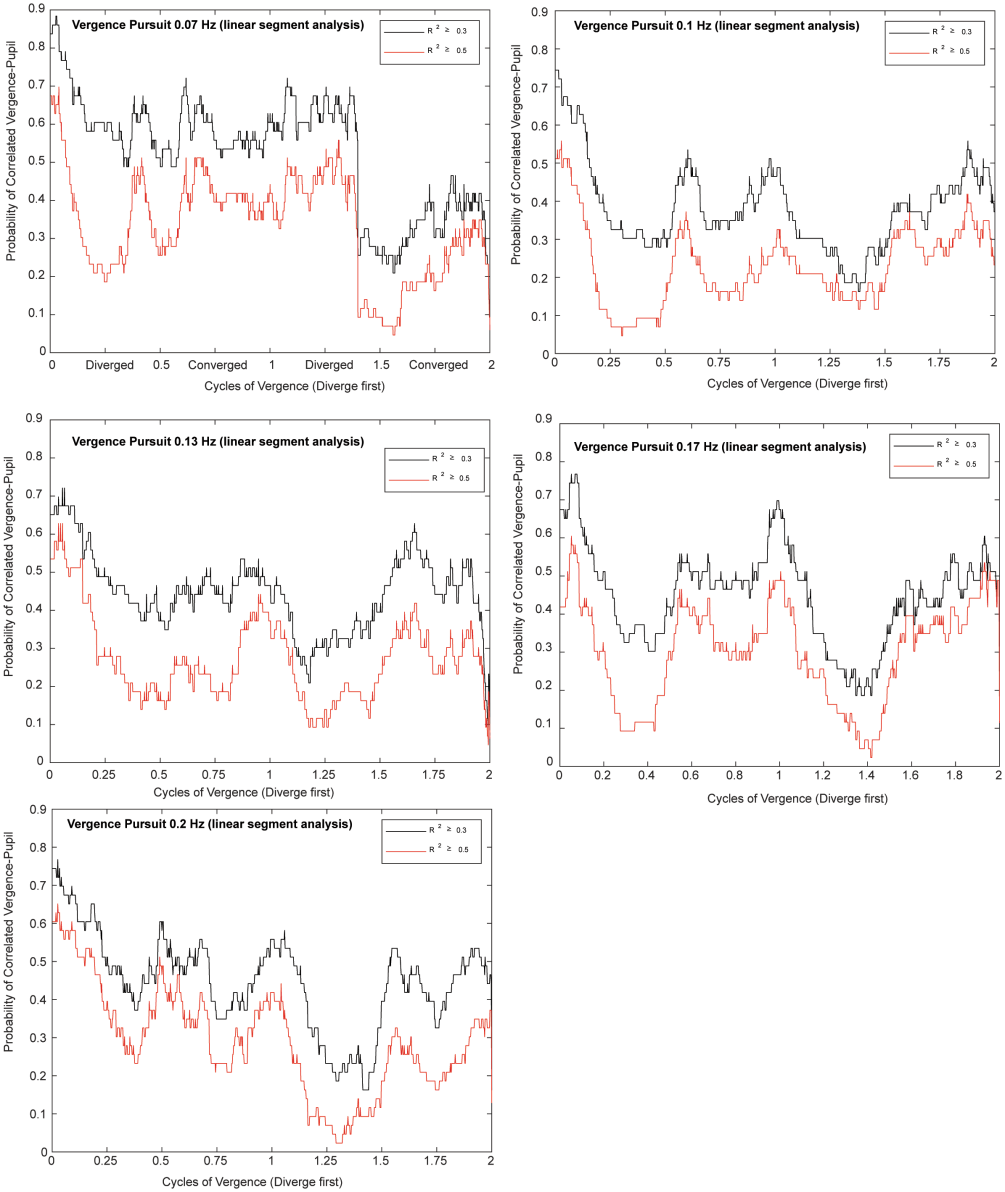
Plots for each frequency of vergence pursuit that show the likelihoods of a linear pupil area-vergence relationship (*R*^2^ ≥ 0.3 in black or *R*^2^ ≥ 0.5 in red) during the response cycles. The traces represent the average likelihood across all subjects for two trials at each frequency. The abscissa is normalized to the cycles of pursuit to illustrate the relationship to the vergence pursuit task. Note the higher likelihood of a linear relationship at vergence pursuit onset, followed periodic fluctuations that are enhanced at middle of the movement range.

**Figure 7:**
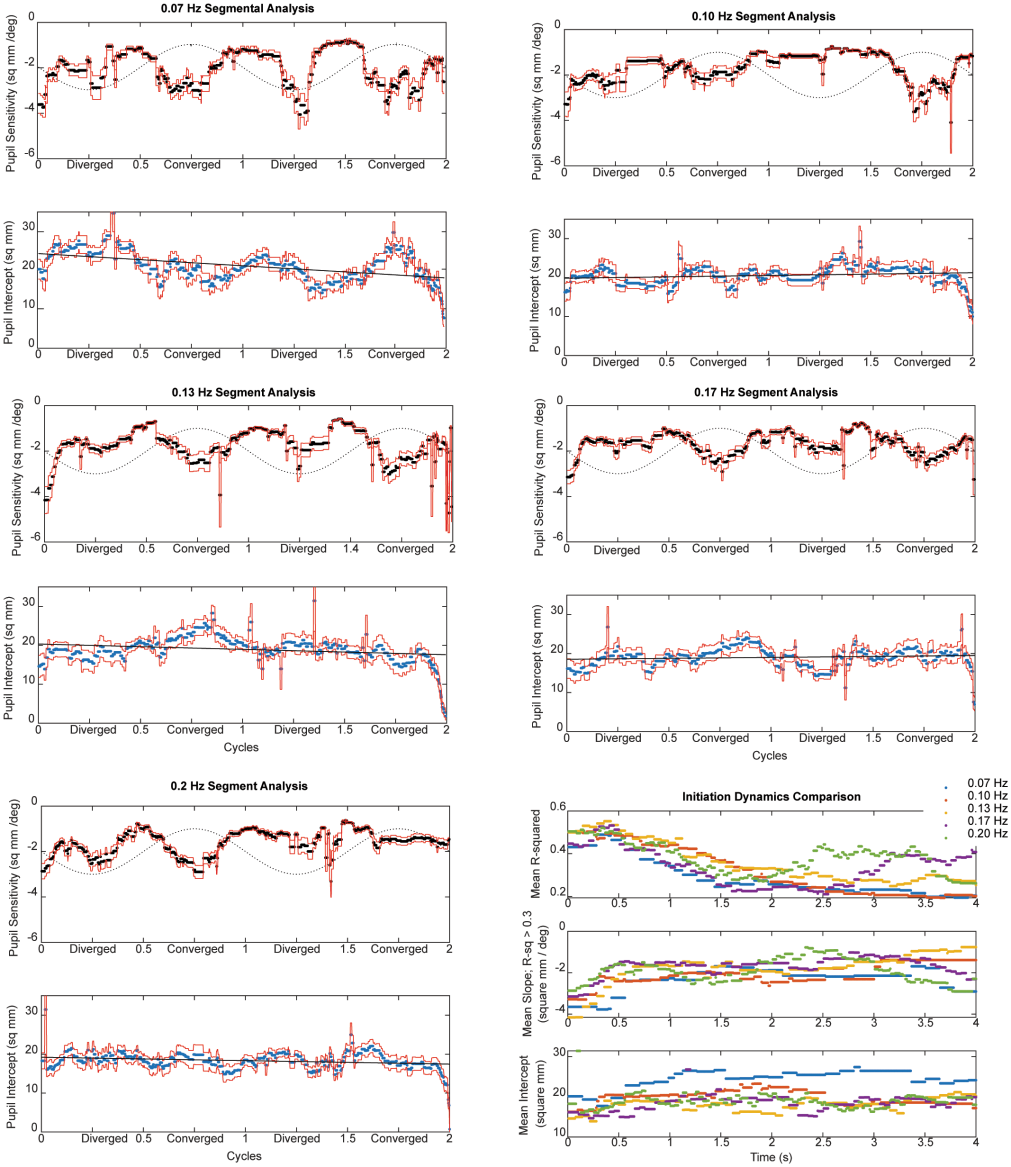
The instantaneous average pupil size sensitivity (in mm^2^/degree vergence) for piecewise linear segments with *R*^2^ ≥ 0.3 across frequencies is shown in the upper panals. The lower panel at each displays the average intercept of the constant term (in mm^2^) across the subjects; the dashed line is a linear regression fit as a function of time. The dashed sine wave in the upper panel schematically shows the target vergence, with convergence represented in the positive direction.

**Figure 8:**
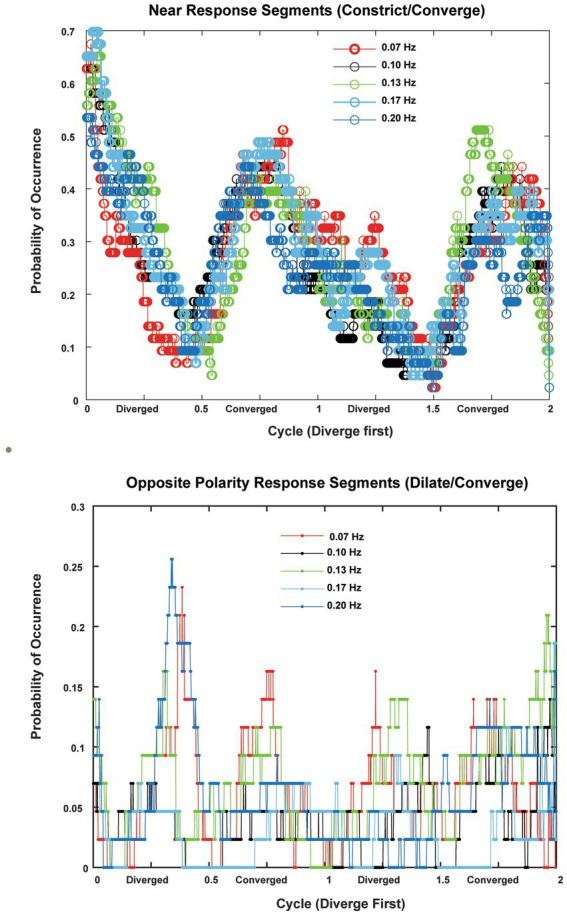
The probabilities of occurrence (in 22 subjects with two trials per frequency) of near response segments (constriction during convergence, dilation during divergence, absolute sensitivity greater than 1 mm^2^/deg. convergence) are shown in the upper panel and the probability of occurrence of opposite polarity responses (constriction during divergence, dilation during convergence, absolute sensitivity greater than 1 mm^2^/deg. convergence). The abscissas are normalized to cycles to show the consistency across responses at 0.07, 0.10, 0.13, 0.17 and 0.2 Hz of disparity pursuit. Binocular disparity is zero prior to the initiation of a trial. Upon initiation of binocular disparity (in the diverging direction), the likelihood of significant ocular divergence with pupil dilation (near response) jumps to a peak value of 0.6–0.7 at all frequencies examined. The opposite polarity responses occurred infrequently across the subjects.

Microscale analysis results are summarized in Figures 5–8. The first analysis viewed the data as a piece-wise linear relationship between pupil area and the vergence angle, with each segment characterized by a pupil sensitivity (slope in mm^2^/degree vergence), a constant (mm^2^ offset) and a goodness-of-fit parameter (*R*^2^). Figure 5 shows that the average *R*^2^ value varied during the disparity pursuit task at each frequency. The goodness-of-fit of line segments is strongest during the first seconds of response initiation and the strength of the linear fits fluctuates with the location of the vergence target during the remainder of each session. At all pursuit frequencies, they tended to be greatest when convergence was near the center of the range for the task (zero or null vergence). Figure 6 shows the same features for the likelihood of a linear pupil area-vergence relationship at *R*^2^ ≥ 0.3 or *R*^2^ ≥ 0.5 during the response cycles. These plots suggest that a transient (onset) response component precedes a steady-state response to pupil-vergence coordination during the smooth tracking task.

Pursuit onset and steady-state response components were prominent in the pupil sensitivity (slope in mm^2^/degree vergence) and constant (mm^2^ offset) estimates from the piecewise linear analysis. For each frequency in Figure 7, the upper panel displays the instantaneous average pupil size sensitivity (in mm^2^/degree vergence) for piecewise linear segments with *R*^2^ ≥ 0.3. The lower panel at each displays the average intercept of the constant term (in mm^2^) across the subjects; the dashed line is a linear regression fit as a function of time. The dashed sine wave in the upper panel schematically shows the target vergence, with convergence represented in the positive direction. As shown in the lower right panel of Figure 7, there is a very similar time course of response initiation during the initial 4 s of the responses across the tested stimulus frequencies of 0.07–0.2 Hz. After this initiation phase, the instantaneous pupil-vergence angle slope magnitudes (mm^2^/deg-vergence) were greatest near peak convergence or divergence, but tended to be greater for convergence at all frequencies. This pattern of pupil responsiveness (re: vergence angle) was very similar across frequencies of binocular disparity pursuit vergence; it is mirrored by the asymmetric constricting versus dilating gains illustrated by the macroscale analysis (Figure 4A). The linear offset/intercept term (lower trace at each frequency) also showed both (a) a baseline drift toward a smaller value and (b) small periodic fluctuations with the vergence angle. Note that the blue line on the baseline drift panels represents the mean baseline drift slope from the macroscale pupil:vergence analysis at that frequency, illustrating analytic consistency of the two approaches.

In the context of the schematic system diagram in Figure 1, the pupil responsiveness over time segments can be either a typical “near response” (constriction with convergence; dilation with divergence) or an opposite polarity response (dilation with convergence; constriction with divergence) (2). The likelihood of these ‘near response polarity segments’ and ‘opposite polarity response segment’ are shown in the upper and lower panels of Figure 8, respectively. The time scales are normalized to cycles of periodic pursuit to show that the variations in likelihood are associated strongly with the degree of convergence of the binocular disparity target for 0.07, 0.10, 0.13, 0.17, and 0.2 Hz of disparity pursuit. Binocular disparity is zero prior to the initiation of a trial. Upon initiation of binocular disparity (in the diverging direction), the likelihood of significant ocular divergence with pupil dilation (near response, upper panel) jumps to a peak value of 0.6–0.7 at all frequencies examined. During this initial stage, there is a low likelihood of an opposite polarity pupil response (lower panel). The likelihood of a near response pattern (upper panel) then declines monotonically as the eyes diverge and then began to converge, reaching a minimum likelihood of about 0.1 immediately before reaching the vergence angle target at the initiation of the trial (baseline). The likelihood then increased monotonically to a peak of about 0.5 as the eyes converge. The pattern then repeats for the second cycle of vergence pursuit, with the likelihood decreasing monotonically from the maximum convergence, through divergence, until the subject displays the vergence angle target at the initiation of the trial (baseline) while the target is again converging.

The occurrence of time segments with an opposite polarity pupil response (constriction with divergence/dilation with convergence, lower panel) was very infrequent and appeared uniformly distributed at most frequencies. However, it showed relative peaks in likelihood at times of maximum divergence or convergence at 0.07 Hz and 0.13 Hz and 0.20 Hz, which is suggestive of a weak ‘range-finding’ strategy to locate a slowly moving binocular disparity target. However, this suggestion needs to be tempered considerably by the low prevalence of these responses.

**Figure 9:**
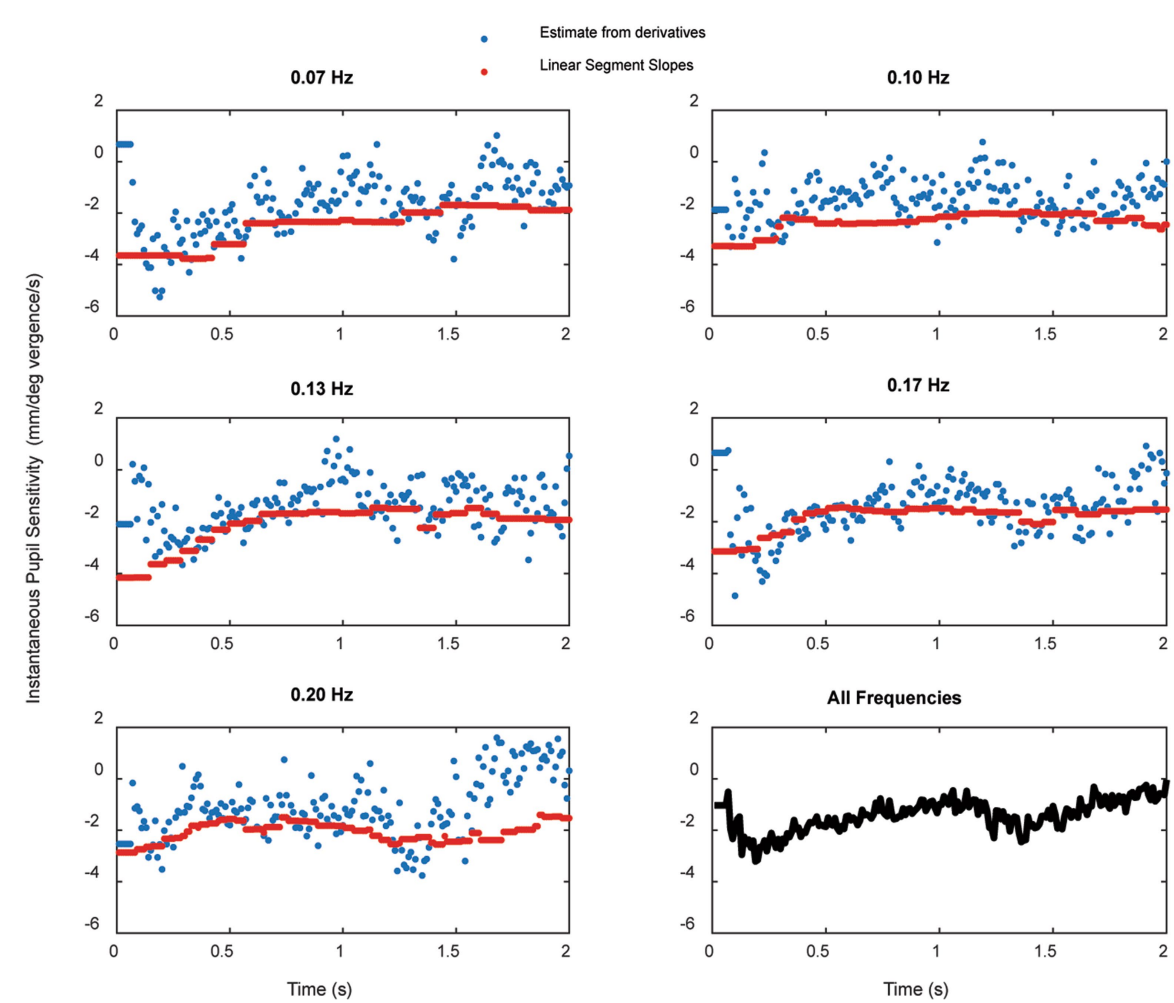
The average instantaneous pupil-to-vergence angle sensitivity (blue) over the first two seconds of stimulation at each frequency. The average slopes from for piecewise linear segments (segment *R*^2^ ≥ 0.3) are plotted in red and the lower right panel shows the grand average across frequencies of the instantaneous pupil-to-vergence angle sensitivity during the first two seconds of the response.

The instantaneous pupil-to-vergence angle sensitivity (d(Pupil Size)/d(Vergence Angle)) was also determined for each subject trial as the ratio of d(Pupil Size)/dt to d(Vergence Angle)/dt at that time point, calculated over a 5 point window (50 ms) centered at each point. Figure 9 shows the average instantaneous pupil-to-vergence angle sensitivity is shown over the first two seconds of stimulation at each frequency in separate panels (blue). The average slopes from for piecewise linear segments (segment *R*^2^ ≥ 0.3) are plotted in red and the lower right panel shows the grand average across frequencies of the instantaneous pupil-to-vergence angle sensitivity during the first two seconds of the response. An initial larger magnitude pupil response sensitivity peaks within 200–300 ms at all frequencies, then reaches a quasi-steady-state within 500 ms of vergence initiation.

**Figure 10:**
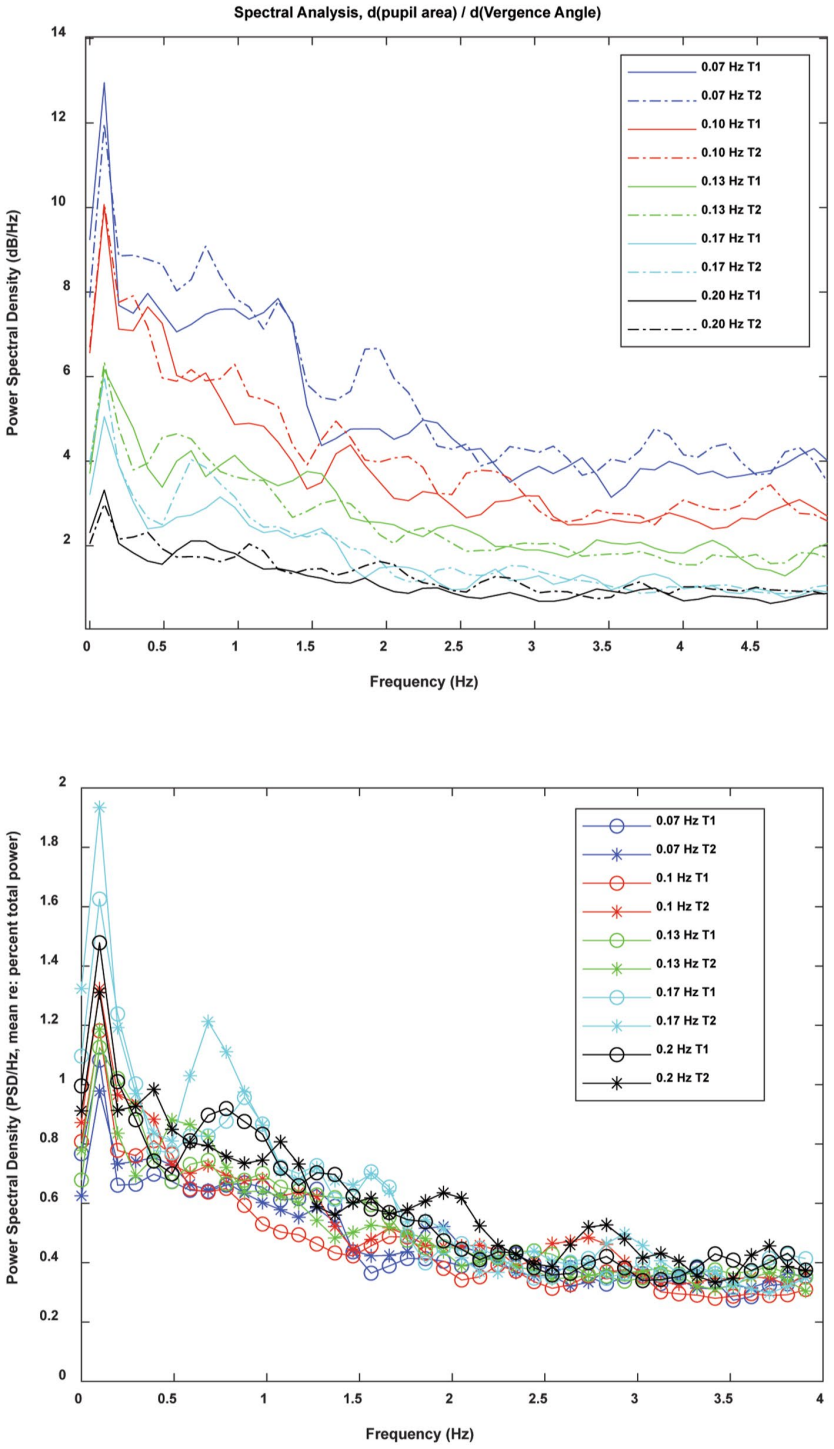
Frequency domain analysis of instantaneous pupil sensitivity to vergence angle during sinusoidal disparity pursuit trials. The raw spectra (upper panel) show the consistency of the spectral content of the instantaneous pupil sensitivity from trials 1 and 2 at each frequency of vergence pursuit. When spectra were normalized for total power (lower panel), the pattern was striking simlar across pursuit frequncies.

In frequency domain analysis, the spectra were highly reproducible across the two trials at each frequency (Figure 10) and had a similar morphology across frequencies. The overall magnitude of the power spectrum also decreased with the target stimulus vergence frequency. There was a peak in power density centered at about 0.1 Hz with a broader plateau in the 0.5–1 Hz frequency range. The raw power density decreased at higher frequencies of fluctuation. When the raw power spectra were represented as percent total power (Figure 10, lower panel), the pattern differed significantly with vergence pursuit frequency in 3 oscillation ranges: (1) the stimulus range (below 0.2 Hz), (2) frequency bands centered in the 0.68–0.89 Hz range, and (3) frequency bands centered in the 1.46–1.66 Hz range. MANOVA followed by Tukey-B post-hoc tests (*p* < 0.05 criterion) indicated that the percent total power of pupil sensitivity fluctutions was significantly lower during 0.07 Hz vergence pursuit than during 0.17 Hz vergence pursuit (Tukey B test, *p* < 0.005). Homogeneous subset analysis indicated that the percent total power of pupil sensitivity fluctutions was between these extremes at other pursuit frequencies, with a higher power at higher stimulus frequencies.

Archival control data sets at 0.1 Hz sinusoidal binocular disparity pursuit trials [see (2, 5)] were added to the new 0.1 Hz trials in order to explore the possibility that there are multiple response patterns in the power spectra. After the 113 individual spectral densities were normalized to reflect percent total power, a fuzzy cluster algorithm [fcm.m in MATLAB, (6)] was applied to test for evidence of distinct spectral density patterns among different groups of subjects. The lack of emergence of separate groups indicates that the spectral content of fluctuations in pupil gain (re: vergence) is homogeneous at that frequency of pursuit.

## Discussion

Coordinated alignment of both eyes begins to develop during infancy and is an important component of accurate three-dimensional visual orientation and information processing. Prism-induced changes in eye alignment and near responses appear in infancy (7, 8) and maturation of vergence and versional binocular motor drive follows during the succeeding 1–3 years. We describe that the dynamic motor control of one class of vergence eye movement, binocular disparity vergence (convergence and divergence) appears to be invariant in normal adults for sinusoidal profiles between 0.07 and 0.20 Hz for stimuli requiring ±1.5° vergence tracking. More than 80% of the variance in the convergence eye movements was explained by a weighted sum of low pass and high pass components, with no significant frequency dependent effects. Although this finding is not surprising, one may suggest that a frequency-invariant control strategy facilitated acquisition and tracking of a broad range of approaching and receding objects in visual space.

Convergence eye movements are accompanied by coordinated movements of the pupil and changes in lens curvature. This coordinated pattern of somatic (vergence eye movement) and autonomic (pupil size regulation) is often termed the near response (1). The “near triad” is a coordinated execution of convergent eye movements, pupillary constriction (miosis) and increased lens curvature. The pupil movements in both eyes are described as consensual. Cross-spectral analysis (a standard signal analytic approach) demonstrates tight consensual fidelity of the left and right pupil responses across frequencies, confirming and quantifying the well-known bilateral symmetry in pupil size regulation. Cross-spectral analysis of the right and left pupil sizes during single trials showed high squared coherence (>0.8) to greater than 3 Hz temporal fluctuations and the cross-spectral phase difference was negligible across the 5 Hz range. This finding provides a quantitative metric of the fidelity of consensual activity across the pupillary dynamic control range in normal subjects.

By contrast, the pupil response metrics (re: vergence angle) that accompanied these vergence eye movements varied with the binocular disparity pursuit frequency. At all frequencies tested, gain in the constricting direction (mm^2^/degree vergence) was of greater magnitude than gain in the dilating direction. These constricted versus dilated fluctuations in sensitivity are even apparent in plots of instantaneous pupil area as a function of instantaneous vergence angle (e.g., Figure 8, lower right panel). Gain magnitudes were lower at 0.07 Hz than 0.13 Hz and did not differ from the latter at higher frequencies. In the constricting direction, it increased to a plateau value at 0.13 Hz. A similar asymmetry (greater velocity during constriction than dilation) was also reported for accommodative pupil responses (9), suggesting that it may be a general dynamic feature of pupil responses. There was also a significant, frequency-dependent per-stimulus linear drift in pupil size, which scaled linearly with the peak binocular disparity velocity across stimulus frequencies. Although we did not identify any age-related effects in vergence or pupil control, our sample size of subjects was much smaller than used the study demonstrating age effects during accommodation (10).

It is important to note that the asymmetric features of co-regulation of pupil area and vergence angle during constriction and dilation have been documented by modeling the pupil response as first-order function of the instantaneous angle across half-cycles of the vergence task. This analytic approach produces a robust fit for these short stimulation periods at one binocular disparity amplitude. However, it does not rule out contributions of higher order dynamics to pupillary movements; it was sufficient for a description of the limited data collected for this study. Longer stimulation epochs at varying disparity amplitudes will permit future refinement of this very basic, first-order model.

The piecewise linear analysis of pupil-vergence coordination indicated that the degree of coupling varied with the vergence angle within a trial. At all frequencies of binocular disparity pursuit, the fidelity of the linear coupling of pupil area to the vergence angle was greatest when the vergence angle was near the initial set point for the binocular disparity trial (i.e., vergence angle for initial binocular disparity stimulus). This position-dependent property was present during both converging and diverging tracking. The microscale analysis indicated that the likelihood of a ‘near response’ pattern (constriction with convergence, dilation with divergence) varied during the task at all frequencies. The behavior was relative to the target cycle. The coupling increased markedly when the eyes were converging from the vergence angle target at the initiation of the trial (baseline) to the maximum convergence of 1.5 degrees. However, it decreased monotonically when the target was moving from maximum convergence to maximum divergence, and continued the same behavior as the target moved (converged back) from maximum divergence to the vergence angle target at the initiation of the trial (baseline). The infrequent opposite polarity pupil response (constriction with divergence/dilation with convergence) was of lower likelihood and more uniformly distributed, with hints of relative peaks at maximum divergence or convergence. These findings suggest three features of controlled coupling of pupil and convergence eye movements within the context of the Figure 1 schema. First, a near response relationship operates with increasing prevalence during convergence (relative to the ‘baseline’ angle); the coupling is higher with increased convergence in this range. Second, the prevalence of ‘near response’-type coupling decreases monotonically in the diverging direction; the decrease persists after the targets move (converge back) from maximum divergence toward the baseline positions, with a minimum prevalence of near response segments near the baseline target position. Third, an opposite polarity pupil response is infrequent, but tends to be more prevalent when the vergence angles are at maximum convergence or divergence for a sinusoidal binocular disparity task. We suggest that the latter response is an exploratory ‘range-validation’ when binocular disparity is relatively constant.

Responses to periodic stimuli typically have an onset phase before they reach a periodic pattern. Pursuit onset and steady-state components of the response were identified by two analyses: (a) piecewise linear characterization of the pupil-size to vergence angle relationship and (b) explicit examination of the instantaneous pupil-to-vergence angle sensitivity, defined as the estimated differential of pupil size relative to the differential of vergence angle. The onset response was an initial period of relatively high pupil:vergence angle sensitivity during the first 500 msec (a divergence), followed by a lower sensitivity steady state response that was reduced during divergence/dilation.

Frequency domain analysis of the estimated differential of pupil area relative to the differential of vergence angle showed two regions of power fluctuations that were sensitive to the pursuit stimulus frequency. These frequency bands centered in the 0.68–0.89 Hz range, and the 1.46–1.66 Hz range, which are within the range of spontaneous, parasympathetic-driven fluctuations termed “hippus” (11). Because power in these bands tended to increase with increasing stimulus frequency, the changes may reflect increased alertness related to cognitive demands for performing the convergence task at higher frequencies/peak velocities of vergence.

The tightly regulated properties of coordinated somatic and visceral motor activity in binocular disparity convergence are consistent with the concept that pupillary aperture selection is a component of visual exploration in three dimensions. These properties in control subjects provide further motivation to refined their utility as differential diagnostic tools in studies of brain injury and neurologic disease (5). Given the indications that pupil activity contains proxy information for attention and cognitive workload (12, 13), it is of interest to note that the pupil activity unrelated to ocular convergence can be identified from the residual after removal of the coupled eye movement-related activity by either our macroscale or microscale analyses (2). This “near-response filtered” pupil activity is worthy of exploration as a measure of non-oculomotor activity.

## Data availability statement

The raw data files supporting the conclusions of this article will be made available by the authors upon request, without undue reservation.

## Ethics statement

The studies involving human participants were reviewed and approved by University of Miami Institutional Review Board. The patients/participants provided their written informed consent to participate in this study.

## Author contributions

CB and MH contributed to the design, data analysis, and writing the manuscript. NN and EW contributed to the study coordination, data collection, and writing the manuscript. AK contributed to the technical aspects of hardware and device software design, both for implementation and in the methods section of the manuscript. All authors contributed to the article and approved the submitted version.

## Conflict of interest

AK was employee by Neurolign USA, Inc. The role of AK was limited to device design and technical aspects of manuscript preparation. Hence, his employment by Neurolign USA, Inc. has no influence on the analysis, discussions, or conclusions of this manuscript.

The remaining authors declare that the research was conducted in the absence of any commercial or financial relationships that could be construed as a potential conflict of interest.

## Publisher’s note

All claims expressed in this article are solely those of the authors and do not necessarily represent those of their affiliated organizations, or those of the publisher, the editors and the reviewers. Any product that may be evaluated in this article, or claim that may be made by its manufacturer, is not guaranteed or endorsed by the publisher.
